# Decoding HMMs using the *k *best paths: algorithms and applications

**DOI:** 10.1186/1471-2105-11-S1-S28

**Published:** 2010-01-18

**Authors:** Daniel G Brown, Daniil Golod

**Affiliations:** 1 Cheriton School of Computer Science, University of Waterloo, 200 University Avenue W., Waterloo, Ontario, Canada N2L 3G1

## Abstract

**Background:**

Traditional algorithms for hidden Markov model decoding seek to maximize either the probability of a state path or the number of positions of a sequence assigned to the correct state. These algorithms provide only a single answer and in practice do not produce good results.

**Results:**

We explore an alternative approach, where we efficiently compute the *k *paths of highest probability to explain a sequence and then either use those paths to explore alternative explanations for a sequence or to combine them into a single explanation. Our procedure uses an online pruning technique to reduce usage of primary memory.

**Conclusion:**

Out algorithm uses much less memory than naive approach. For membrane proteins, even simple path combination algorithms give good explanations, and if we look at the paths we are combining, we can give a sense of confidence in the explanation as well. For proteins with two topologies, the *k *best paths can give insight into both correct explanations of a sequence, a feature lacking from traditional algorithms in this domain.

## Background

Hidden Markov model (HMM) decoding is a basic problem in sequence analysis, as HMMs are used throughout the field to divide discrete sequences into regions corresponding to features. HMMs decode a sequence by assigning each position in the sequence a label; intervals with the same label then correspond to the same feature in a sequence.

Two common decoding procedures for HMMs are the Viterbi and posterior algorithms. The Viterbi algorithm computes the maximum probability path through an HMM, and returns the labelling of the states of that path. Many states may share a single label. While it is easy to compute this single path, it often gives poor annotations [1]. We might want to compute the labelling of a sequence that has maximum probability, but that is *NP*-hard [2]. Posterior decoding computes the most probable state or label of each position of a sequence and joins those together into a single labelling. This is quickly computable, but has no guarantee that it will actually correspond to a feasible labelling of the sequence, since it may not satisfy the constraints of the model.

Recently, variant posterior methods have also appeared, which seek to maximize the expected number of positions in the sequence that are correctly labelled, or the geometric mean of the probability that a position is correctly labelled, while requiring that a labelling has nonzero probability [3,4]. However, the overall labelling may be of extremely low probability relative to the true explanation; again, it is *NP*-hard to maximize the overall labelling probability [2]. Also, several heuristic algorithms, such as the 1-best by Krogh [1], exist to work around these limitations, but do not guarantee optimality.

We study an alternative approach: we investigate the feasibility of computing the *k *most probable paths, and how examining the labellings corresponding to these paths can serve as a good alternative to more traditional HMM procedures. We have used the HMM for the Phobius [5] transmembrane topology predictor to investigate the usefulness of the *k*-best paths in a real world example and show that alternative paths can provide a wealth of information. We show that *k*-best paths can be good predictors and investigate ways to extract such predictions. We also show how to judge our confidence in a particular prediction by looking at the alternatives that result from the *k*-best paths.

Finally, we investigate the use of the *k*-best paths to predict more then one topology in cases when it is biologically proven that such alternatives exist.

### Notation and definitions

A hidden Markov model is a probabilistic generative automaton that produces a sequence while traversing stochastically through a finite set of states. An HMM is defined by a collection of parameters: a start state *I*, a set of transition parameters (*a*_*ij*_ ), and a set of emission parameters (*b*_*i*_(*c*)). Let *m *be the number of states. A path *π *through the HMM is a sequence of states *π*_0 _= *I*, *π*_1_, *π*_2_, ..., *π*_*n *_such that for all *i*, . For each path *π *of length *n *+ 1 the probability that this path emits sequence *x *= *x*_1_*x*_2 _... *x*_*n *_is . The Viterbi algorithm finds the most probable path through the HMM for a given sequence. The natural implementation of the Viterbi algorithm (see [6]) uses dynamic programming to construct a Θ(*mn*) sized matrix, in which cells correspond to state-position pairs. There is a natural extension to finding the *k*-best paths in the HMM: store the *k *highest-scoring paths for each position-state pair. The *k *highest probability paths for *x*_1_... *x*_*i *_that end in state *π*_*i *_must be from the *k*-best paths to each of the states for the sequence from *x*_1 _to *x*_*i*-1_. This observation leads to a Viterbi-like algorithm with runtime *k *times as large, and requiring Θ(*kmn*) storage. This approach has been used in speech recognition as early as the 1990s [7], but its space requirements make it infeasible for finding the *k*-best paths for large values of *k *on substantial HMMs for long sequences.

Alternatively, finding the *k*-best paths though an HMM can be seen as finding *k *shortest paths in a graph with *nm *nodes (one for each position-state pair). The graph has *O*(*m*^2^*n*) edges, each edge corresponding to a potential transition between HMM states. On this graph, one can apply Eppstein's algorithm [8] to find *k *shortest paths in *O*(*m*^2^*n *+ *k*) time. This algorithm keeps the whole Θ(*m*^2^*n*)-size graph in memory as well as an Ω(*mn*) size structure used in the algorithm; it also has high constant factors in the runtime and only implicitly represents the paths.

**Figure 1:**
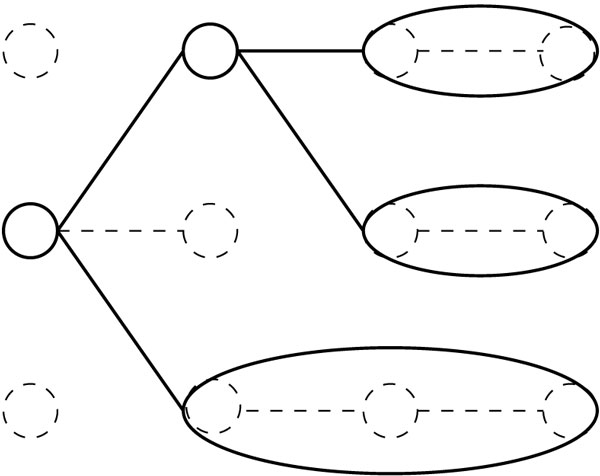
**Example of compressed tree**. Solid nodes and lines indicate the actual data structure. The dotted nodes and lines indicate intermediate steps in the construction described in the text.

A space savings approach for Viterbi was presented by Sramek *et al*. [9], which uses a compressed tree approach to actively free memory used by unnecessary back pointers in the state-position matrix. We create an *m *by *n *+ 1 grid of nodes where each node corresponds to a cell of the Viterbi matrix (a position-state pair): each column corresponds to the *m *possible choices for the last state in a prefix of *x*. We create an edge between node *v*_*i *_of column *i *and node *v*_*i*_+1 of column *i *+ 1 if the Viterbi path to the position-state pair (*i *+ 1, *v*_*i*+1_) goes through *v*_*i*_. Edges give the back pointers in the dynamic programming for the Viterbi calculation. If we remove all nodes and edges that are not on paths from column *n *of the graph to state *I *in column 0, what remains is a tree. In the approach of Sramek *et al*., this tree is actively pruned, and nodes with exactly one parent are merged into their parents as they are created. An example of a compressed tree is in Figure 1. After compression, each node corresponds to a sequence of states which emits a particular substring of the given string, found in a potential Viterbi path to one of the leaves of the tree. For details of how to maintain a compressed tree efficiently see [9,10].

## Methods

The Viterbi algorithm (and procedures for finding the *k*-best paths) actually consists of two different calculations: computing the probabilities of the best path (or *k *paths) to every state for every prefix *x*_1_... *x*_*i *_of a given sequence *x*_1_... *x*_*n*_, and also storing the pointers necessary to reconstruct those paths. Our primary emphasis is to store enough information to reconstruct these pointers for the final paths in as little space as possible, again using an active pruning procedure, which becomes a bit more complicated with *k *paths than with just one. Using this method, we typically use much less memory than the Θ(*kmn*) required by the obvious approach.

### Computing the probabilities

To compute the *k *highest path probabilities to each state at position *i*, we assume that we have a sorted list of the *k*-best path probabilities to each state at position *i *- 1. Then, if we are considering a state *a *whose possible predecessors in the HMM are *Pred*(*a*), we can find the *k*-best probabilities for state a at postion *i *by performing an operation very similar to the first *k *steps we would undertake in merging |*Pred*(*a*)| lists for mergesort. The Viterbi probability of the ℓ-th best path to state *v *is

We can compute this quantity in *O*(*m*) time per path; it is an interesting algorithmic question whether this can be sped up heuristically, since all paths to state *v *that were in state *c *at position *i *- 1 will have their probabilities multiplied by the same constant, *a*_*cv *_*b*_*v*_(*x*_*i*_).

This calculation, then, takes *k *times the cost of a standard Viterbi calculation and Θ(*mk*) space. By contrast, the posterior algorithms yielding non-zero probability paths of Fariselli *et al*. [4] and of Kall *et al*. [3] run asymptotically as fast as Viterbi, but yield only a single path, which may be overall an odd one from a macroscopic view.

### Storing and pruning the paths

The *k *best paths can be computed by using a three-dimensional grid, where entry (*i*, *j*, *a*) corresponds to the *a*-th best path to state *j *for sequence *x*_1_, ..., *x*_*i*_. Treating this grid as a graph, we draw an edge from (*i*, *j*, *a*) to (*i *+ 1, *k*, *b*) if the *b*-th best path to state *k *at position *i *+ 1 uses the *a*-th best path to state *j *at position *i*. As in the Viterbi algorithm, we wish to actively maintain only the entries in the graph that correspond to paths to the current frontier, position *i *of the sequence.

**Figure 2:**
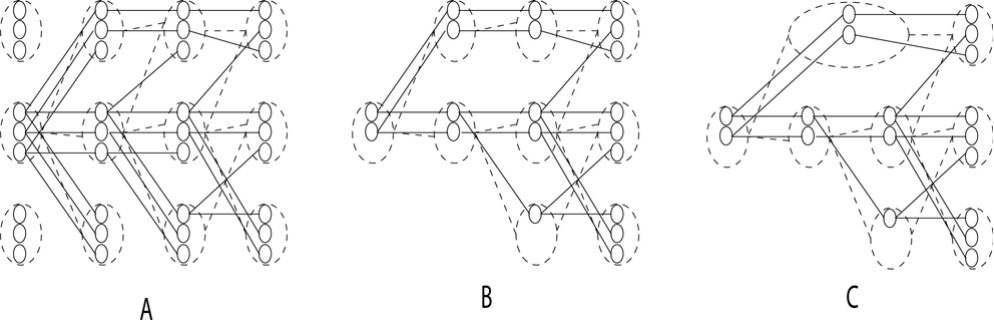
***K*-best tree construction visualization**. The solid lines represent path level nodes and edges, while dotted lines represent vertex level nodes and edges. A) The tree structure before any pruning or compressing. B) The tree structure after path and state nodes are removed. C) The final data structure, after all node merging.

We will describe two types of nodes in the graph: path nodes, which correspond to a single value of (*i*, *j*, *a*), and state nodes, which correspond to all paths (*i*, *j*, *a*) where *i *and *j *are kept constant (see Figure 2A). An edge between two state nodes exists if any of their path nodes have an edge.

As we move forward in the sequence, we compute the *k *best paths to each state at a new position *i*, drawing edges in the structure to the predecessor of each. Then, we prune and compress. First, we must prune away all of the path and state nodes from the previous level that are no longer found on paths to a leaf (and all nodes orphaned by that change, until none remain to be pruned); see Figure 2B. Then, we merge pairs of consecutive state nodes for which all paths from one state node go to the other and vice versa; see Figure 2C.

Each path node stores its number of children and pointers to its state node, and the path node that is its parent. Each state node stores the list of HMM states associated with it (the state path through the HMM for the sequence interval corresponding to that state node), the list of at most *k *path nodes it includes, pointers to its children, and the number of state nodes that are its parents. We can find the parents of a state node by examining its associated path nodes and identifying the state node corresponding to the parent of each path node.

A state node is deleted if it has no path nodes, and it is merged with another state node if that node is its only parent and it is the parent's only child. This corresponds to the situation where all paths that include a sequence of states *π*_*i*_... *π*_*j *_for the subsequence *s*_*i*_... *s*_*j *_are followed by the same set of states *π*_*j*__+1 _... *π*_*k *_for *s*_*j*__+1 _... *s*_*k*_, so we can merge the sequences together.

#### Pruning and compressing details

Suppose we are about to incorporate sequence letter, *x*_*i*_. We calculate the probability of all *k *best paths to each possible state. If the ℓ-th best path to state *c *uses path node (*i'*, *b*, *a*) at the previous step (where *i' *need not be *i *- 1 due to compression), then we set that node to be the parent of (*i*, *c*, ℓ), updating the child counters for (*i'*, *b*, *a*), and we also set the state node (*i'*, *b*) to have (*i*, *c*) as a child.

After performing this set of operations for all the new graph nodes corresponding to sequence position *i *(at all states), we prune all nodes not reachable from the new leaves, by seeing which leaves at level *i *- 1 are no longer accessible. For each path node in this "removal list", we remove the path node from its state node, and update appropriate counters. If its parent's child counter reaches zero, it is moved to the removal list as well. If a path node removed was the last path node for that state node, then the state node is removed. We also detect if a state node enters the condition that it has only one child and its child has only that one parent: if so, the states are merged.

Because this algorithm is done online, the active footprint of memory used by the algorithm is dramatically less than Θ(*kmn*) in practice, though it may be that large in extreme cases.

### Recovering the paths

Once we have produced the final structure, we must extract the *k *paths with highest probability. The *k *path probabilities at each of the *m *leaves are the probabilities of the best paths to those states. From these *km *paths, we select the *k *of highest probability, as the first *k *steps of an *m*-way mergesort, in *O*(*km*) time, and we construct the *k*-best paths then in *O*(*kn*) time after the merging. The total run time is *O*(*nm*^2^*k*) as for the naive algorithm, as the overhead in doing the paths compression is smaller then the basic calculations. The is no guarantee that the new procedure is more space-efficient: our heuristic may not always result in compression.

## Results and discussion

Having *k *different high-probability explanations for a sequence might offer some assistance in decoding it. Here, after briefly showing that our pruning and compression methods make finding the *k *best paths for large values of *k *possible, we explore several different uses for these multiple explanations: first, to see if any of them is a good explanation, second, to see if we can reconstruct a good single explanation from a set of paths, third, to see if the topologies and probabilities assigned by the HMM to the *k *best paths help us identify easy and hard sequences to decode, and finally (and perhaps most interestingly) to see if *k *paths can help us decode sequences with different *true *explanations.

Our experiments use the domain of transmembrane protein topology prediction, where Viterbi-style decoding has not been successful. We have used the 188-state HMM from Phobius [5], which divides alpha-helical membrane protein sequences into segments corresponding to membrane-spanning segments and the parts found either inside or outside the cellular membrane. The *topology *of a membrane protein is the number of the membrane-spanning helical regions, along with the *sidedness*, which is the identification of the first residue of the protein as being either intracellular or extracellular.

We use the data set of proteins provided with Phobius [5]: 247 membrane proteins with no signal peptide and 45 proteins with a signal peptide. We focus on the larger of these two sets. Note that Kall *et al*. evaluated cross validated data sets, making direct comparisons to their published results inappropriate. We also note that their HMM has been trained for success with the 1-best algorithm, while we are using a quite different decoding approach. In our last experiment, we study five proteins known to have two topologies [11].

A fundamental question for this study is what makes a good prediction. Transmembrane topology prediction is somewhat imprecise because the actual boundaries of membrane-spanning segments are inexact, but can be identified to within a residue or so based on solved protein structures [12]. The authors of Phobius describe a prediction as correct if it identifies the correct topology, and if each true helix overlaps with the corresponding helix in at least five positions. Helices tend to be approximately twenty-two residues long, so this measure is lax.

We also studied a more stringent correctness measure, parameterized by a tolerance *τ*. In it, a prediction is correct if topology is correct, and if all predicted helix boundaries are no more than *τ *residues away from the true boundary. We used this measure with values of *τ *from 0 to 5 in our experiments.

### Memory and runtime

Our algorithms do dramatically reduce the memory use of finding the *k *best paths in this HMM. Table 1 shows the maximum memory required in decoding the 45 proteins with a signal peptide for different values of *k*: while these values do grow with *k*, the memory usage for the tree-based approach is approximately fifty times smaller than for the naive approach; it takes twice as much memory as an approach that only computes the probabilities, and does not store back pointers at all. We note that in this application, we could have used the naive algorithm in the memory footprint of a typical computer. However, we also computed the 10, 000 best paths for the dual-topology proteins described below, which would not have been possible with the naive algorithm [13].

**Table 1:** Overall memory usage. Overall memory usage required to process all proteins with a signal peptide in Mb.

	Probability only	Tree-based	Matrix-based
*k *= 100	4	8	290
*k *= 200	6	13	570
*k *= 300	7	17	850
*k *= 400	8	21	1130

This value corresponds to the memory required for the longest protein. 'Probability only' corresponds to a run where no backtracking was performed, 'Tree-based' refers to our tree-based implementation, 'Matrix-based' refers to naive matrix approach.

Meanwhile, Table 2 shows the runtimes for these three approaches: our algorithm is the slowest, but the overhead required for pruning and compression has the effect of doubling the runtime of the more naive methods.

**Table 2:** Total runtime. Amount of time required to process all proteins with a signal peptides, in seconds.

	Probability only	Tree-based	Matrix-based
*k *= 100	84	145	90
*k *= 200	161	303	166
*k *= 300	231	425	241
*k *= 400	296	553	317

### Finding at least one good labelling

Now, we explore the *k *best paths to see if any of them gives a good labelling. We compare with the results of the 1-best algorithm [1], decoding algorithm for which the model is trained. Our results are in Table 3. There is much information in the *k*-best paths; the challenge is in extracting this into a single prediction. For example, we find a good labelling in the set of 100 best paths much more often than in the single labelling found by the 1-best algorithm. It is also striking that for 46 of the 247 proteins (19%), the exactly correct labelling is found among the top 1000 paths.

**Table 3:** Proteins with good labellings. Proteins with good labellings.

	*τ *= 0	*τ *= 1	*τ *= 2	*τ *= 3	*τ *= 4	*τ *= 5	Phobius
*k *= 1 (Viterbi)	2	11	19	27	46	59	137
*k *= 10	9	21	33	44	67	83	157
*k *= 100	34	52	64	85	110	129	198
*k *= 1000	46	66	89	123	143	168	214

1-best	4	18	26	38	62	79	166

For *k *paths and a correctness measure, we count the number of proteins for which at least one of the top *k *paths gives a prediction that satisfies that correctness measure. The bottom line gives the results for the 1-best decoding algorithm used in Phobius. Presented is the data set without signal peptide (*n *= 247).

### From many labellings to one

Once we have identified the top *k *paths, the next challenge is in summarizing them into a single labelling. We divide the *k *paths into "groups", where all paths in a group predict the same topology, and then form a consensus from the heaviest group, for which the sum of the conditional probabilities of all of the chosen paths in that group is highest. Note that this method cannot succeed when the chosen group is not, in fact, of the correct topology.

Many natural ways to form a consensus all gave essentially equivalent results. For example, we might average the positions of start points and end points of transmembrane helices for all paths in a group. This approach always produces consistent results. This method is fairly good at retrieving the information contained in the group, as shown in Table 4. For the Phobius correctness measure, at most four proteins for which the largest group of *k *paths gave the correct topology were mis-annotated, after building the consensus (data not shown). In general, this straightforward approach to moving from *k *paths to a single labelling did less well than the 1-best algorithm, but better than Viterbi, though the results are much closer for our *τ *= 5 measure then for the Phobius measure.

**Table 4:** Forming a consensus labelling. Forming a consensus labelling from *k *paths. Results for Viterbi decoding and 1-best decoding (as used in Phobius) are provided for comparison. (*n *= 247).

	*τ *= 0	*τ *= 1	*τ *= 2	*τ *= 3	*τ *= 4	*τ *= 5	Phobius
*k *= 10	3	12	23	27	47	69	139
*k *= 100	4	14	23	32	51	70	141
*k *= 1000	3	15	24	35	57	77	145

Viterbi (*k *= 1)	2	11	19	27	46	59	137
1-best	4	18	26	38	62	79	166

Other approaches to forming a consensus, such as allowing per-position voting among the *k *paths on the correct label of a position (which is in some sense analogous to posterior decoding), or allowing predictions to vote on the start position and length of helices yielded similar results. Both of these methods can yield labellings inconsistent with the model after generating a consensus.

### Using *k *paths to increase confidence

Another potential use of multiple paths is to reinforce our belief that a particular protein is easy or hard to properly annotate.

In particular, we hypothesized that if the top *k *paths all correspond to the same number of helices (with possible differences in sidedness or in the positions of helix boundaries), this can be seen as supporting evidence for that number of helices. Indeed, this is confirmed by the results in Table 5. If the top *k *paths all give the same number of helices, this prediction is very likely correct, and in the majority of cases, the full topology is also correctly chosen. Such proteins almost always form a good consensus.

**Table 5:** Gaining confidence. If the top *k *paths all agree about the number of helices, this prediction is correct in 83% to 91% of cases. If they disagree, then in only 55% to 71% of cases does the largest group predict the correct helix number. A similar separation occurs for the overall protein topology.

	All same number of helices			Distinct number of helices		
**Type of correctness**	***k *= 10**	***k *= 100**	***k *= 1000**	**1*k *= 10**	***k *= 100**	***k *= 1000**
	**(*n *= 189)**	**(*n *= 120)**	**(*n *= 64)**	**(*n *= 58)**	**(*n *= 127)**	**(*n *= 183)**

# of helices	156(83%)	107(89%)	58(91%)	32(55%)	81(64%)	130(71%)

Overall topology	123(65%)	93(77%)	56(88%)	19(33%)	51(40%)	93(51%)

By contrast, for the proteins where multiple different numbers of helices are predicted, the results are much weaker: the largest group of predictions gives the correct number of helices in 20% to 28% fewer cases, and gives the overall correct topology in 32% to 37% fewer cases.

Another use for *k *paths is to identify proteins for which the top *k *paths use up a significant part of the conditional probability space of the model, given the sequence. If so, then we hypothesize that their consensus labelling is likely to be good. This hypothesis is confirmed, as shown in Table 6. Consider the 47 proteins where the top 100 paths take up more than conditional probability of 0.5 given the sequence. For 38 of them (81%), at least one of those paths satisfies our correctness measure with *τ *= 5. By contrast, for the 102 with total probability of the top 100 paths less than 0.01, only 31 (30%) have a path this good. Thus, the total conditional probability of the top *k *paths is a good predictor of the existence of paths with a good labelling among these *k *paths.

**Table 6:** Correct proteins as a function of conditional probability. Number of proteins predicted correctly as a function of the total conditional probability of the top *k *paths. Results are shown for the data set without signal peptide (*n *= 247), with correctness being measured with *τ *= 5.

Probability of top *k *paths	*k *= 10		*k *= 100		*k *= 1000	
	
	Correct	Incorrect	Correct	Incorrect	Correct	Incorrect
< 0.01	33	98	31	72	33	45
0.01 - 0.5	42	64	60	37	82	27
0.5 - 1	8	2	38	9	53	7

### Two true answers: dual-topology proteins

An interesting final testbed for our ideas are proteins known to have two topologies; such *dual-topology *proteins were only confirmed to exist in 2006 by Rapp *et al*. [11]: the five proteins identified are EmrE [Swiss-Prot:P23895], SugE [Swiss-Prot:P69937], CrcB [Swiss-Prot:P37002], YdgC [Swiss-Prot:P0ACX0], and YnfA [Swiss-Prot:P76169].

For all of these proteins, the two topologies differ only in their *sidedness*: they all are short proteins with four transmembrane helices, and have very little information in their non-membrane segments to indicate which set belong inside or outside the membrane.

The Phobius model, by itself, does not give a good prediction for most of these proteins: the topology from Uniprot [14] is approximated by any of the top 100 paths in the model in two of five cases. One challenge for these proteins is that because they are quite short, the signal peptide module in the Phobius model often gives them a bad labelling; if we remove that module, then even the top ten paths gives this one correct topology in four of five cases (data not shown).

However, much more interesting is the question of finding two good answers. If we look at the top 100 or 1000 paths, then for all of these proteins, the top paths support two or more different topologies. In three cases, superficially YnfA, CrcB and SugE, the two heaviest groups do give both correct overall topologies, and the consensus of the these paths is correct for the Phobius distance measure. For the other two proteins, EmrE and YdgC, the top two groups are not correct: in both cases one of the two groups incorrectly predicts three helices, not four.

For proteins with two (or more) correct topologies, the top *k *paths let us explore that space effectively. We expect that similar explorations may also be fruitful in other contexts where multiple correct answers are to be found. We did not investigate the question of how often proteins with a single topology might appear to have two topologies using a similar approach.

## Conclusion

We have developed a memory-efficient algorithm for finding *k*-best paths in an HMM. Considering the *k *best paths of an HMM is not novel; the idea has been considered by speech recognition experts, for example [7]. However, previous algorithms for this have either used too much memory or been heuristic in nature. Our method has a significantly lower memory footprint in practice than the naive implementation. Using this algorithm we investigated the use of *k*-best paths in topology prediction for transmembrane proteins. While better than the Viterbi algorithm, forming a consensus of the *k*-best paths does not perform as well as the 1-best algorithm; largely, the issue is in finding the correct overall topology, which 1-best does better, possibly for training reasons. Where the *k*-best paths gives the overall correct topology, we can almost always compute a good consensus prediction. We can extract other interesting data from the *k*-best paths. In particular, we can estimate our confidence in a prediction by looking at the content and probability distribution of the *k*-best paths. Finally we have shown that in the special case of dual-topology proteins, a simple processing of the *k*-best paths can often predict both of the correct topologies of a protein.

## Competing interests

The authors declare that they have no competing interests.

## Authors' contributions

DB conceived of the study, wrote most of the manuscript, and directed the experiments. DG designed most of the algorithms, wrote some of the manuscript, conducted the experiments, and did much of the evaluation.
